# Acoustofluidic Biosensors

**DOI:** 10.3390/mi17050561

**Published:** 2026-04-30

**Authors:** Chun-Jui Chen, Jae-Sung Kwon, Han-Sheng Chuang

**Affiliations:** 1 Department of Biomedical Engineering, National Cheng Kung University, Tainan 701, Taiwan; s8390116@gmail.com; 2 Department of Mechanical Engineering, Incheon National University, Incheon 22012, Republic of Korea; jsungkwon@inu.ac.kr; 3 Convergence Research Center for Insect Vectors (CRCIV), Incheon National University, Incheon 22012, Republic of Korea; 4 Nuclear Safety Research Institute, Incheon National University, Incheon 22012, Republic of Korea; 5 Medical Device Innovation Center, National Cheng Kung University, Tainan 701, Taiwan

**Keywords:** acoustofluidic, biosensors, surface acoustic wave, point-of-care, biomarker

## Abstract

The rapid and precise detection of biomarkers and pathogens remains a critical challenge in clinical diagnostics. Traditional methodologies are frequently hindered by protracted workflows, complex sample preparation, and reliance on resource-intensive instrumentation. Acoustofluidics—the synergistic integration of acoustics and microfluidics—has emerged as a transformative solution for point-of-care testing (POCT). Bulk acoustic wave (BAW) and surface acoustic wave (SAW) technologies enable the contactless, label-free, and biocompatible manipulation of bioparticles across micro- and nanometer scales. This review critically examines recent advancements in BAW- and SAW-based acoustofluidic biosensors. We elucidate the fundamental principles governing distinct acoustic modes—including Quartz Crystal Microbalance (QCM), film bulk acoustic resonator (FBAR), and Solidly Mounted Resonator (SMR) for BAW and Rayleigh and Love waves for SAW—and evaluate their specific roles in liquid-phase sensing, particle sorting, and cellular focusing. Results show that integrating on-chip sample preparation accelerates diagnostic workflows, reducing assay times to under 10 min. Coupling acoustic manipulation with optical, mass-based, or electrochemical modalities effectively overcomes fundamental diffusion limits, achieving ultrasensitive, multimodal detection. We address translational challenges—acoustothermal heating, biofouling, and scalable integration. Following a discussion of clinical applications in oncology and infectious diseases, we map emerging trajectories, emphasizing AI-driven intelligent microfluidics, modular architectures, and flexible wearable platforms that will ultimately democratize continuous precision diagnostics.

## 1. Introduction and Background

The increasing demand for precision medicine and point-of-care testing (POCT) has accelerated the development of rapid, highly sensitive, and portable biosensors [1]. Although gold-standard methods such as ELISA and PCR offer exceptional diagnostic accuracy, their reliance on complex, multistep protocols and centralized laboratory infrastructure limits their deployment in field settings [2]. Various active microfluidic technologies—including dielectrophoresis (DEP), rapid electrokinetic patterning (REP), magnetophoresis, and optical tweezers—have been explored to address these challenges. However, these methods face inherent operational constraints: DEP and REP are highly relatively to medium conductivity (>1 mS/m), magnetic manipulation typically requires time-consuming prelabeling with functionalized beads, and optical tweezers, despite their high spatial precision, suffer from low throughput and the potential to induce photothermal damage in delicate biological samples [3,4,5]. By contrast, acoustofluidics provides a label-free, contactless, and highly biocompatible alternative. By operating at power intensities comparable with routine clinical ultrasound, acoustic platforms can efficiently manipulate targets within native biofluids (e.g., whole blood and saliva) without compromising structural integrity or inducing thermal degradation, offering a robust and versatile platform for integrated biosensing [6,7,8].

The development of acoustofluidics originated from bulk acoustic wave (BAW) systems in macroscale chambers, establishing the fundamental theoretical principles of acoustic manipulation, most notably the acoustic radiation force (ARF) theories formulated by King and Gor’Kov [9,10]. Early experiments, such as the Kundt tube, successfully achieved macroscopic particle levitation; however, their clinical translation was hindered by large device footprints and excessive power consumption (>1 W), which induced acoustothermal heating. During the 1990s, the field shifted from macro-acoustics toward the emergence of miniaturized Micro Total Analysis Systems (MicroTAS). Although MicroTAS helped democratize lab-on-a-chip diagnostics, these passive devices encountered a significant kinetic hurdle known as the “diffusion limit.” Their sole reliance on slow molecular diffusion often extends assay times to 60–120 min, falling far short of the rapid turnaround (<15 min) required for effective POCT triage. In addition, processing complex samples, such as plasma or saliva, in passive microchannels often leads to rapid biofouling and nonspecific binding (NSB) [11,12,13,14].

To overcome these kinetic and matrix limitations, modern acoustofluidics has evolved through the synergistic integration of physical acoustics with microfluidic platforms. This integration particularly favors shear-horizontal surface acoustic waves (SH-SAW) and Love waves, which minimize acoustic energy leakage and preserve high Q-factors in liquid environments. This architecture exploits the exquisite mass sensitivity of piezoelectric resonators, where target binding induces measurable shifts in resonance frequency [15,16,17]. The active driving of fluid and particle motion enables the integration of up to four synergistic functions on a single chip, which can be tailored to specific diagnostic needs. For rapid assay kinetics, acoustic streaming generates convective microvortices that rapidly homogenize reagents and reduce reaction times from hours to minutes. When target enrichment is required, ARF can concentrate entities—ranging from macromolecules to whole cells—into high-density regions, effectively amplifying local analyte concentrations. For sample purification, acoustic washing employs controlled hydrodynamic drag forces to selectively disrupt weak, nonspecific bonds, dramatically improving signal-to-noise ratios (SNRs) in complex biofluids [18,19]. Moreover, specific acoustic parameters can trigger sonoporation, inducing transient membrane permeabilization to facilitate reagent-free intracellular access and lysis [20,21].

The monolithic integration of these synergistic forces has driven significant clinical translation. The recent AIMDx platform demonstrates technological progress by using acoustic purification and streaming-induced lysis to detect SARS-CoV-2 RNA and host antibodies directly from raw saliva in under 30 min [22]. In oncology, platforms such as ASCENDx use SAW-induced vortices to concentrate exosomes for noninvasive cancer monitoring [23]. Other significant advances include ultrarapid SAW biosensors for HIV diagnosis [24], tear-based LCN1 screening for diabetic retinopathy [7,8], and cardiovascular markers such as ApoB and CRP [15]. By overcoming kinetic limitations and providing laboratory-level sensitivity, acoustofluidic platforms effectively realize the MicroTAS vision.

**Figure 1:**
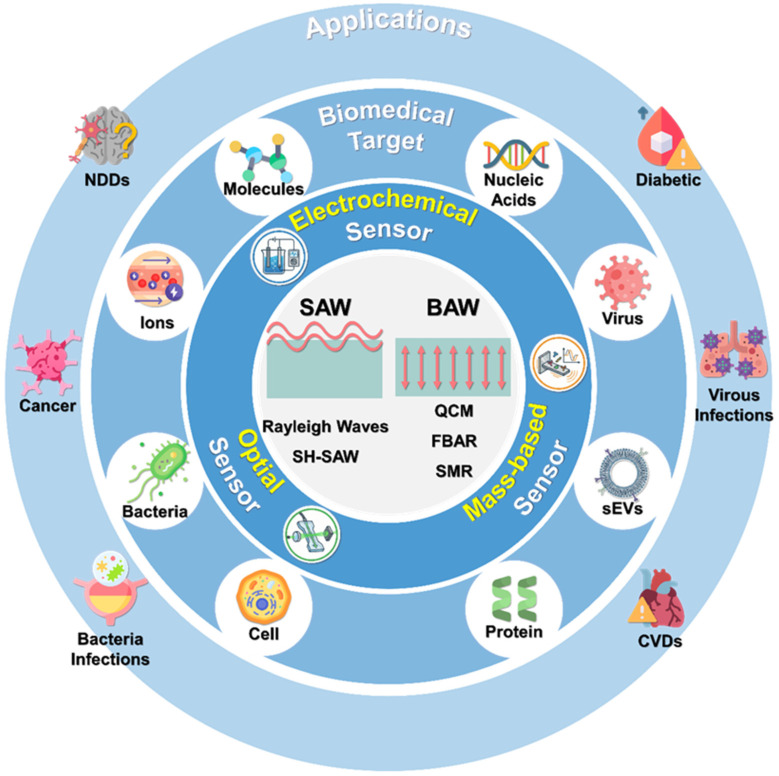
A comprehensive framework of acoustic-wave-based biosensing: From fundamental resonators to clinical applications.

To systematically evaluate the progress in this rapidly expanding field, this review is organized into a four-layer hierarchical framework (Figure 1). The innermost layer examines the fundamental physics of acoustic waves, distinguishing between SAW and BAW mechanisms. The second layer categorizes the predominant transducer and sensor architectures, including mass-based, optical, and electrochemical modalities. Moving outward, the third layer delineates the multiscale biomedical targets manipulated by these systems, ranging from small-molecule biomarkers to whole cells. Finally, the outermost layer highlights disease-oriented translational progress, demonstrating how acoustofluidic biosensors are being developed to address critical diagnostic challenges in infectious diseases, oncology, metabolic disorders, cardiovascular conditions, and neurodegenerative diseases. Beyond current applications, this review addresses the persistent translational challenges hindering field deployment—acoustothermal heating, biofouling, and scalable integration. Furthermore, we explore emerging technological frontiers, including AI-powered intelligent microfluidics, hybrid multimodal sensing, and the potential of flexible wearable devices for point-of-care clinical diagnostics.

## 2. Design Principles, Physical Mechanisms, and Integrated Sample Preparation

### 2.1. Substrate and Transducer Fundamentals

Acoustofluidic systems are broadly categorized into two primary modalities depending on their wave propagation physics: SAW and BAW. The intrinsic properties and operational parameters of various piezoelectric substrates utilized in these technologies are summarized in Table 1 and Table 2.

SAW characteristics are fundamentally governed by the crystal cut and wave propagation direction, with acoustic waves typically excited via interdigital transducers (IDTs) patterned directly onto the piezoelectric substrate [25]. The Rayleigh SAW (R-SAW) mode is commonly employed for acoustofluidic manipulation and gas sensing because of its strong surface displacement and high electromechanical coupling. For instance, the 128° Y-X LiNbO_3_ cut has traditionally served as an industry standard because of its high electromechanical coupling coefficient (4–6%), which promotes efficient actuation [26]. ST-cut quartz provides an ultralow temperature coefficient of frequency (TCF), ensuring excellent thermal stability [27,28]. High-coupling cuts, such as 41° Y-X LiNbO_3_, produce substantially large effective coupling, while a 152° Y-rotated LiNbO_3_ cut reduces in-plane anisotropy for omnidirectional SAW propagation [29,30]. A fundamental limitation of R-SAWs is their severe acoustic attenuation in liquid environments; their out-of-plane vertical displacement directly radiates energy into the fluid, rendering them suboptimal for liquid-phase biosensing [31].

Shear-horizontal SAW (SH-SAW) modes are preferentially utilized to overcome the above acoustic damping. Their particle displacement is predominantly parallel to the surface, largely suppressing energy radiation into the contacting liquid [17]. Common SH-SAW substrates include 36° Y-X LiTaO_3_ and 64° Y-X LiNbO_3_, which provide strong surface wave confinement [32]. Langasite (LGS) exhibits a near-zero TCF and maintains thermal stability above 1000 °C, and potassium niobate (KNbO_3_) supports pure SH-SAWs with exceptionally high coupling [33]. Despite these advantageous features, traditional bulk SAW sensors face inherent challenges. Materials such as LiNbO_3_ and LiTaO_3_ are highly susceptible to temperature fluctuations, leading to thermal signal drift [34]. In addition, the rigidity, brittleness, and high manufacturing costs of traditional bulk piezoelectric substrates restrict their integration into affordable, disposable, or conformal wearable platforms [35,36].

BAW devices utilize thickness-mode vibrations within piezoelectric substrates sandwiched between two metallic electrodes. These devices generate standing acoustic waves capable of sensing minute mass accumulations or manipulating suspended bioparticles [37]. BAW devices are primarily classified into three structural architectures: quartz crystal microbalance (QCM), film bulk acoustic resonator (FBAR), and solidly mounted resonator (SMR). For low-frequency applications, QCMs typically employ AT-cut quartz operating in the thickness-shear mode, which ensures excellent temperature stability and chemical resistance [38,39,40]. However, a critical limitation of QCMs is that their physical scaling bound for enhancing mass sensitivity necessitates thinning the quartz wafer, thereby rendering the device mechanically fragile and impeding miniaturization for high-density array integration [39,41].

To access the gigahertz (GHz) frequency regime, FBARs utilize suspended ultrathin piezoelectric films. Aluminum nitride (AlN) is widely preferred for these films because of its high acoustic velocity and CMOS compatibility. Furthermore, alloying AlN with scandium significantly enhances its electromechanical coupling coefficient from a few percent to over 12% [42,43]. Zinc oxide (ZnO) provides strong piezoelectricity and excellent biocompatibility, and lead zirconate titanate (PZT) boasts the highest coupling coefficients but suffers from substantial acoustic losses and poor biocompatibility [44,45]. While FBARs deliver ultrahigh sensitivity, they face engineering challenges related to their fragile suspended membranes and severe acoustic energy dissipation in liquids, particularly when operating in longitudinal modes [46].

To mitigate the mechanical fragility inherent to suspended films, SMRs replace the air cavity with a Bragg reflector stack—comprising alternating layers of high- and low-acoustic-impedance materials—to strictly confine acoustic energy within the active piezoelectric region [43]. This configuration yields a mechanically robust structure that is highly resilient for liquid-phase operations and amenable to wafer-scale integration. Nevertheless, fabricating the multilayer reflector stack requires highly precise and controlled deposition processes, which can increase manufacturing time and costs [47].

Flexible polymeric substrates such as polyethylene naphthalate and polyimide have emerged as highly attractive alternatives to circumvent the mechanical stiffness and economic barriers of traditional crystalline materials. Sputtering thin piezoelectric films (e.g., ZnO or AlN) onto these polymers yields flexible devices capable of generating high-frequency acoustic fields, including Lamb waves. This structural flexibility enables conformal attachment to human skin, propelling the development of disposable wearable biosensors for continuous sweat pH monitoring and noninvasive pathogen detection [35,48]. Despite this immense potential, the development of acoustic sensors on soft polymers remains nontrivial. Primary bottlenecks include severe acoustic energy dissipation into the viscoelastic substrate, suboptimal crystallinity of the piezoelectric layer when deposited on amorphous polymers, and interfacial adhesion failure driven by thermal expansion mismatches. Furthermore, the ultrathin form factor frequently excites multiple interfering acoustic modes, severely complicating phase and signal demodulation for precision biosensing [49,50].

**Table 1:** Comparison of common piezoelectric substrates for SAW generation.

Category	Mode	Materials	Selection Considerations	Drawbacks & Challenges	Ref.
SAW	R-SAW	128° Y-X LiNbO_3_	High coupling efficiency. Standard for driving acoustic streaming.	High Temperature Sensitivity. Prone to breaking during thermal processing.	[51]
		152° Y-X LiNbO_3_,	Quasi-Isotropic Propagation.	Lower K^2^ compared to the 128° Y-X LiNbO_3_. Higher material costs.	[29]
		ST-cut Quartz	Extreme Frequency Stability.	High signal loss. Needs more electrodes.	[51]
		41° Y-X LiNbO_3_	Highest Coupling Coefficient.	High propagation loss. Unsuitable for long-distance.	
	SH-SAW	36° Y-X LiTaO_3_,	Gold Standard for biosensing. Temperature stability. High Q-factor in liquids.	Lower power efficiency. Requires higher voltage.	
		64° Y-X LiNbO_3_	High Sensitivity.	High Temperature Sensitivity.	
		41° Y-X LiNbO_3_	Highest Coupling Coefficient.	Parasitic bulk wave radiation.	
		LGS	Environment Stability. Zero-TCF cuts available for high-precision stability.	Lower power efficiency Higher material costs.	[52]
		KNbO_3_	Ultra-sensitive mass detection. High-velocity waves.	Extremely fragile. Prone to damage.	[53]

**Table 2:** Comparison of common piezoelectric materials and configurations for BAW devices.

Acoustic Wave Category	Mode	Materials	Selection Considerations	Drawbacks & Challenges	Ref.
BAW	QCM	AT-cut Quartz	High thermal stability Low liquid damping	Low coupling efficiency. Lower mass sensitivity.	[54]
		LGS	Extreme thermal stability Superior in liquids	Difficult to process into ultrathin membranes for high sensitivity.	[52]
	FBAR	AlN	CMOS-compatible & chemically robust. High velocity & Q-factor	Low coupling efficiency High stress & cracking	[55]
		ScAlN	Boosts coupling efficiency with AIN	Lower stiffness & Q-factor Non-uniform properties	[42]
		ZnO	High coupling & easy synthesis Highly biocompatible	Non-CMOS compatible Chemically & thermally unstable:	[44]
		PZT	High electromechanical coupling capabilities,	Toxic/Poor biocompatibility High acoustic attenuation	[56]
	SMR	Alternating high/low impedance materials at the bottom	Robust physical structure. Excellent acoustic confinement	Costly and time-consuming. Difficult stress control Minor acoustic leakage	[57]

### 2.2. Interdigital Transducers Fundamentals and Geometry

**Figure 2:**
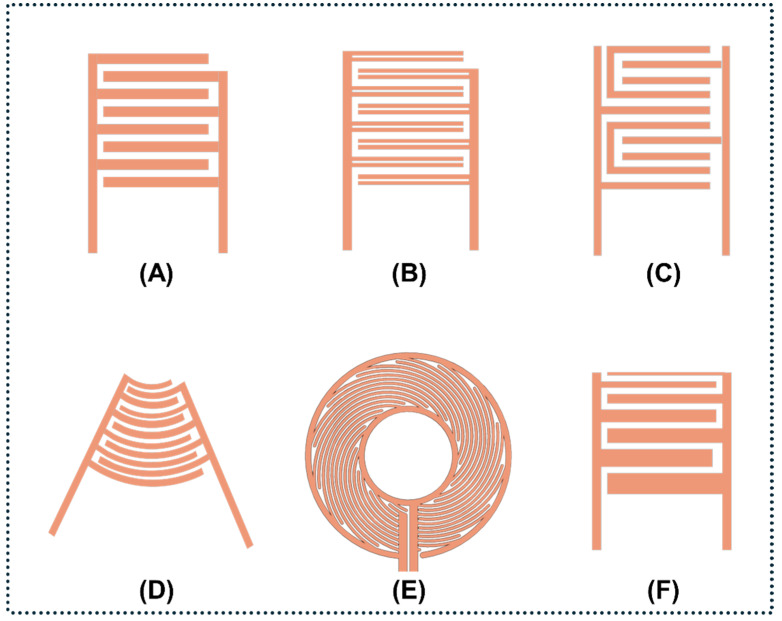
Schematic of IDT structures. (**A**) Straight IDTs; (**B**) Split-finger IDTs; (**C**) FEUDTs; (**D**) FIDTs; (**E**) Spiral IDTs; (**F**) Chirped finger IDTs.

The IDT is the core electroacoustic component of SAW devices, converting radiofrequency (RF) electrical signals into mechanical acoustic waves via the inverse piezoelectric effect. Electrode periodicity defines the acoustic wavelength (λ), and critical parameters—such as aperture size, number of finger pairs, and electrode materials (e.g., Al for high acoustic velocity, Au for bio-inertness)—must be meticulously optimized to ensure energy efficiency and spatial confinement [7]. The geometric arrangement of the IDT strictly governs the spatial distribution and functional characteristics of the generated acoustic field, as illustrated by the diverse architectures in Figure 2.

**Straight IDTs** feature uniform electrodes (typically *λ*/*4*) that deliver robust, broadband fundamental modes. This configuration is ideal for power-intensive bulk processing and large-scale acoustic streaming [58].
**Split-finger IDTs** utilize narrow electrodes (typically *λ*/*8*) that explicitly suppress mechanical reflections and minimize acoustic energy loss, yielding the high-Q resonances required for precision biosensing [59].
**Floating electrode unidirectional transducers** incorporate electrically unconnected (floating) electrodes between active fingers to alter the surface electrical loading. This modification promotes forward-wave transmission, significantly reduces insertion loss, and enables the high-frequency operations critical for ultrasensitive biosensing [60,61].
**Focused IDTs** employ curved or concentric arcs to concentrate acoustic energy into microscale focal points, maximizing acoustic intensity for high-speed, precision tasks such as fluorescence-activated cell sorting without excessive input power [62].
**Spiral IDTs** utilize circular or spiral layouts to generate omnidirectional SAWs. When applied to sessile droplets, these transducers induce symmetric azimuthal streaming that functions as an on-chip microcentrifuge, thereby enabling rapid submicron particle enrichment [63].
**Chirped IDTs** vary the finger pitch across the transducer to create continuous frequency gradients. This design enables dynamic acoustic beam steering via RF signal sweeps, supporting real-time, size-dependent continuous-flow fractionation [64].
**Chirped IDTs**: Vary finger pitch to create continuous frequency gradients. This enables dynamic acoustic beam steering via RF signal sweeps, supporting real-time, size-dependent continuous-flow fractionation [65].

### 2.3. Acoustic Wave Mode

#### 2.3.1. Acoustic Force Mechanisms

The object movement within an acoustofluidic system is governed by a dynamic physical balance among the primary acoustic radiation force (F_pARF_), secondary acoustic radiation force (F_sARF_, or interparticle Bjerknes force), acoustic streaming-induced fluidic drag, and thermoviscous effects. These mechanisms are scale-dependent and fundamentally dictated by the acoustic wave modes (e.g., standing vs. traveling waves) and thermal boundary conditions [8,65,66].

F_pARF_ is influenced by the characteristics of the acoustic field. In the standing surface acoustic wave (SSAW) mode, the force arises from the pressure gradient. The periodic nature of SAW mode enables the trapping of analytes at acoustic pressure nodes or antinodes. The migration direction of a particle within this standing wave is dictated by its acoustic contrast factor (Φ), a dimensionless parameter governed by the relative density and compressibility between the particle and suspending fluid. Particles with a positive contrast factor (Φ > 0, e.g., most mammalian cells and rigid microbeads) are driven toward the acoustic pressure nodes, whereas “acoustically soft” particles (Φ<0, e.g., lipid vesicles and elastomeric particles) are trapped at the antinodes [37,67]. By contrast, the traveling surface acoustic wave (TSAW) mode does not possess fixed wave nodes. Instead, it generates continuous unidirectional thrust. The magnitude of TSAW force is modulated by the acoustic radiation force factor ($Y_{T}$), which is strongly dependent on the particle’s intrinsic material properties (i.e., acoustic impedance), thereby enabling the highly selective sorting of identical sized particles. The acoustic radiation forces of SSAW and TSAW modes are fundamentally governed by the particle’s intrinsic material properties (i.e., density, compressibility, and acoustic impedance), allowing the selective sorting of particles of identical sizes but different mechanical phenotypes (e.g., through contrast factor variations in SSAW or nonlinear scattering resonances in TSAW) [68,69].

Interparticle interactions are dominated by F_sARF_, which exist in SSAW and TSAW fields. Given that the strength of F_sARF_ scales is inversely proportional to the fourth power of the interparticle distance ${(F}_{sARF}\propto {d_{C}}^{-4})$, dispersed particles experience negligible interference when they are far apart. Once the particles are initially concentrated into high-density regions, secondary forces take over, forming high-density clusters from the loose particles [19,70,71].

The progressive wave (TSAW) is primarily characterized by a dimensionless parameter, kappa factor $\kappa =\pi d_{p}/\lambda$ ($d_{p}$: particle diameter; λ: wavelength). When κ>1, momentum transfer-driven F_sARF_ predominates; when κ<1, particles follow the acoustic streaming. For SSAW, the transition is governed by a critical particle diameter ($D_{c}$), which is determined through the force equilibrium between the volume-dependent F_pARF_ ${(F}_{rad}\propto {d_{p}}^{3})$ and the diameter-dependent acoustic streaming drag force ${(F}_{drag}\propto d_{p})$ [7,8,65,72,73].

When the particle diameter $d_{p}>D_{c}$, ARF dominates, causing particles to be captured or pushed toward pressure wave nodes. When $d_{p}<D_{c}$, streaming drag prevails, and particles tend to follow the circulating flow vortices, leading to mixing. Meanwhile, Dc∝f−12. Therefore, increasing the frequency (f) can effectively reduce $D_{c}$ to a submicrometer scale, enabling the direct capture of nanosized targets such as exosomes [65,74,75].

The operating range of the acoustic system is limited by the attenuation length ${(x}_{s}={\rho_{s}c}_{s}\lambda_{s}/\rho_{f}C_{f})$. $x_{s}$ determines the width of the microchannel, preventing acoustic waves from attenuating before reaching the distal end of the channel, which could lead to an uneven distribution of the force field within the channel [8,76,77]. The ability of sound flow to overcome the diffusion barrier can be quantified by calculating the Péclet number $\left(Pe_{ac}=u_{str\;}L/D\right)$, where $u_{str}$ is the acoustic streaming velocity, L is the characteristic length, and D is the diffusion coefficient. When $Pe_{ac}>100,$ acoustic convection dominates; when $Pe_{ac}<0.1,$ diffusion dominates. Compared with SSAW, the Eckart flow induced by TSAW generally exhibits a significantly higher $Pe_{ac}$, which can substantially reduce the incubation time of biosensors [78]. Furthermore, significant thermoviscous coupling exists between the transport efficiency ($Pe_{ac}$) and thermal boundary layer ($\delta_{t}$). Local temperature rises when the thermal boundary layer decreases in fluid viscosity, leading to a synergistic enhancement in acoustically driven convection transport and $Pe_{ac}$ and thereby effectively shortening the saturation detection time [79,80]. However, excessive thermal accumulation may deactivate biological receptors on the sensor surface, posing a trade-off between sensitivity enhancement and biological stability [79,81].

#### 2.3.2. Rayleigh Surface Acoustic Waves

**Figure 3:**
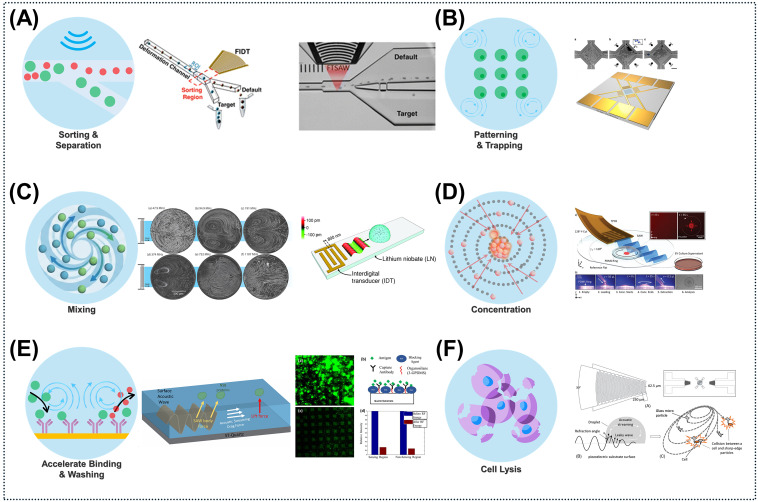
Multifunctional acoustofluidic applications. Acoustic forces allow precise control in three areas: 1. Particle manipulation: (**A**) Rapid, high-viability single-cell sorting using focused traveling surface acoustic waves (FTSAW) [86]. (**B**) High-resolution 2D patterning with acoustic potential wells created by orthogonal SSAW [90]. 2. Fluid dynamic enhancement: (**C**) Quick in-droplet mixing that surpasses diffusion limits via high-frequency SAWs [97]. (**D**) Acoustofluidic centrifuge for ultrafast concentration of extracellular vesicles [98]. 3. Bio-interface optimization: (**E**) Acoustic washing to selectively remove interfering NSB proteins [95]. (**F**) Use of focused SAW and glass beads to generate centrifugal forces for lysing *Candida albicans* cells, achieving efficient lysis within 5 min to support DNA amplification [99]. “Figures reprinted with permission (Ref. [86] Copyright 2023 Lab on a Chip; Ref. [90] Copyright 2015 Nature Communications; Ref. [97] Copyright 2014 Advanced Materials; Ref. [98] Copyright 2023 Small; Ref. [99] Copyright 2024 Wiley Analytical Science)”.

R-SAWs exhibit elliptical particle motion with out-of-plane (vertical) and in-plane (longitudinal) displacement components that decay exponentially into the substrate [8,82,83,84]. At solid–liquid interfaces, the vertical component strongly couples to the fluid, radiating compressional longitudinal waves and causing significant attenuation of the surface waves—a phenomenon known as “leaky SAW” [31,72]. Although this severe energy dissipation may hinder high-resolution gravimetric sensing, the efficient energy transfer generates strong ARF and acoustic streaming flow (ASF), which are exceptionally well suited for advanced acoustofluidic actuation. As illustrated in Figure 3, R-SAWs support diverse applications across three main domains: precise particle manipulation, enhanced fluid dynamics, and optimized bio-interface interactions.

ARF enables the contactless, label-free manipulation of bioparticles ranging from 100 nm to 30 μm, with the exerted force increasing in direct proportion to particle volume [84,85]. This highly nonlinear relationship provides robust size selectivity, as a twofold increase in particle diameter results in an eightfold increase in acoustic force. In SSAW fields, particles with a positive acoustic contrast factor (Φ > 0) migrate toward pressure nodes, allowing continuous flow configurations to achieve highly efficient size-based separation and sorting. For example, the use of focused traveling SAWs in image-activated sorting enables the continuous separation of living circulating tumor cells (CTCs) within microfluidic devices [86,87]. Furthermore, dynamic sorting via frequency or phase modulation combined with ring-resonator tweezers creates high-Q acoustic “cages”. These structures enable the stable trapping and patterning of rare cells from large sample volumes [88,89] and the assembly of highly controlled 2D single-cell arrays [90].

For submicron targets where the ARF is relatively weak, ASF emerges as the dominant manipulation mechanism. Streaming-induced steady flows and microvortices effectively overcome Brownian diffusion limits, enabling rapid fluid homogenization—such as ultrahigh-frequency (UHF) nanoliter-droplet mixing [91]. Furthermore, ASF actively transports nanoparticles to sensor surfaces to accelerate binding kinetics, a strategy that proves highly effective when synergized with metal-enhanced fluorescence [92]. This active enrichment effectively combines acoustic manipulation with selective capture modules to isolate low-abundance targets [93]. On this basis, acoustofluidic centrifuges leverage SAW-driven vortices to rapidly concentrate nanoparticles and exosomes within 0.12 s, obviating the need for conventional, resource-intensive ultracentrifugation and achieving the ultrafast enrichment of rare targets [93]. Omnidirectional spiral surface acoustic wave architectures extend this capability by inducing 360° symmetric azimuthal rotation, resulting in uniform particle concentration independent of size variations [63]. In addition, an acoustofluidics-assisted bimodal fluorescence/SERS biosensor utilizes SAWs to concentrate nanobeads and exosomes (30–150 nm) at the stagnation point of a microcapillary. Requiring a minimal sample volume of merely 0.5 µL, the platform completes on-chip fluorescence detection within 15 s and exhibits significantly enhanced signal sensitivity [19]. The intense mechanical shear forces generated by these acoustic waves can also be harnessed to rupture cell membranes for reagent-free, on-chip cell lysis [51].

Beyond sample preparation, ASF provides a highly reliable methodology for biosensor washing and surface regeneration. High-frequency acoustic actuation produces localized shear stresses sufficient to disrupt weak van der Waals interactions. This dynamic washing continuously removes loosely bound molecules while preserving specific covalent biorecognition bonds [94]. For instance, the application of R-SAWs successfully eliminated up to 86% of NSB antigens and 94% of interfering background proteins, significantly amplifying the SNR in complex clinical biofluids. Moreover, the intense acoustic energy derived from amplified RF signals can intentionally disrupt specific antigen–antibody bonds, enabling the rapid regeneration of sensor surfaces [95]. When coupled with chemical dissociation buffers, this acoustic actuation facilitates robust device reusability across multiple diagnostic test cycles [96].

Despite these advantages, the primary operational limitations of R-SAWs stem from their shallow acoustic penetration into the fluid phase. This shallow coupling leads to rapid power dissipation and localized acoustothermal heating, posing a risk of denaturing temperature-sensitive bioreceptors. Even with these thermal constraints, the powerful combination of ARF-driven and ASF-induced mass transport establishes R-SAWs as a highly versatile and foundational technology for acoustofluidic biosensors.

#### 2.3.3. Shear-Horizontal SAW and Love Waves

**Figure 4:**
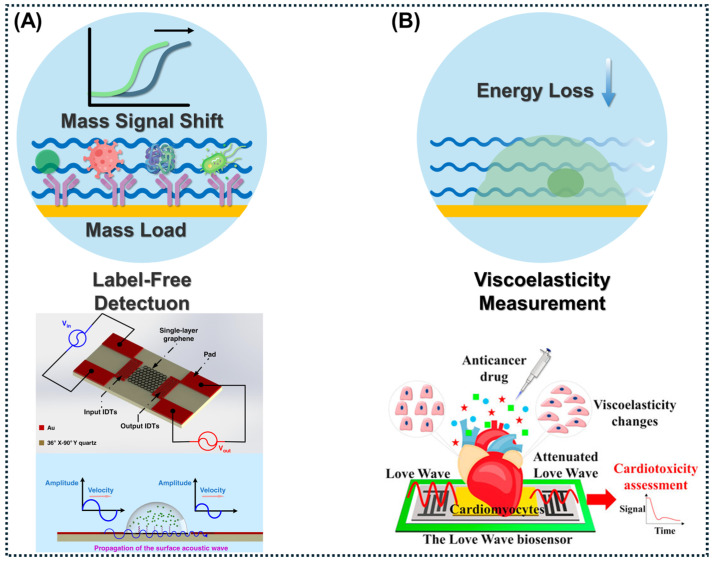
Mechanisms and applications of SH-SAW biosensors in biomedical detection. 1. Label-free detection: Utilizes the mass loading effect. 2. Monitoring cell dynamics: Enables the real-time tracking of target behavior. 3. Viscoelasticity measurement: Measures energy loss and wave attenuation caused by targets. (**A**) Prostate-specific antigen (PSA) detection: Highly sensitive microfluidic Love-wave (a type of SH-SAW) chip integrated with aptamer probes [102]. (**B**) Evaluating doxorubicin (ADM)-induced cardiotoxicity by measuring the viscoelasticity of cell populations [105]. “Figures reprinted with permission (Ref. [102] Copyright 2020 Microsystems & Nanoengineering; Ref. [105] Copyright 2021 Journal of The Electrochemical Society)”.

SH-SAWs exhibit particle displacement parallel to the substrate surface and perpendicular to the direction of wave propagation [100]. This specific transverse polarization prevents the excessive radiation of compressional acoustic energy into contacting liquids, significantly reducing acoustic damping and rendering SH-SAWs highly effective for liquid-phase biosensing. Common piezoelectric substrates optimized for generating SH-SAWs include 36° YX-cut LiTaO_3_, 64° YX-cut LiNbO_3_, and ST-cut quartz. For further improvement in surface sensitivity, SH-SAWs are frequently converted into Love waves by depositing a thin acoustic guiding layer (e.g., SiO_2_, ZnO, PMMA, or SU-8) over the propagation path. For the effectively formation of Love waves, the shear acoustic velocity of this guiding layer must be lower than that of the underlying piezoelectric substrate [101]. This acoustic mismatch traps and confines the wave energy within the top sensing layer while shielding the IDTs from conductive biofluids. Under optimized conditions—specifically regarding guiding-layer thickness, operating frequency, mode purity, and specific density/viscosity of liquid loading—Love-wave architectures can demonstrate exceptionally high gravimetric sensitivity. This feature makes them highly advantageous for label-free detection in complex biological matrices, such as the highly sensitive quantification of endotoxins using an aptamer-based SH-SAW biosensor integrated with a single-layer graphene film [102] as Figure 4A and the selective real-time acoustic detection of cancer biomarkers [103,104].

Beyond simple mass loading, the phase shift and insertion loss of SH-SAWs can be utilized to precisely determine the viscoelastic properties of the contacting medium, allowing the accurate measurement of mechanical variations at functionalized biological interfaces [105] Figure 4B and the dynamic monitoring of complex fluids such as human plasma [106]. Alternatively, SH-SAWs can be generated alongside conventional Rayleigh waves using specialized IDT designs and dual-line configurations. These hybrid devices combine the strong acoustic streaming of Rayleigh modes for rapid sample mixing with the low-loss liquid-phase operation of SH-SAWs, enabling efficient particle manipulation and highly sensitive biosensing on a single monolithic platform [78]. The sensitivity of SH-SAWs to surface-acoustic loading also enables the noninvasive monitoring of cellular dynamics, as demonstrated successfully through the real-time tracking of tendon stem cell adhesion on parylene-C-coated sensors and the continuous assessment of dynamic cell spreading in in vitro wound healing assays [17,107,108].

#### 2.3.4. Bulk Acoustic Wave

**Figure 5:**
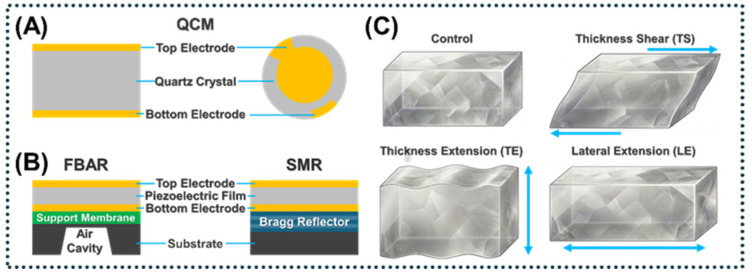
(**A**) Design of a typical QCM. (**B**) Design of a typical FBAR and diagram of a SMR. (**C**) Schematic of propagation modes (TS/TE/LE).

The performance of BAW devices is inherently tied to their propagation physics and mode selection (Figure 5). Different from SAWs, BAWs establish standing acoustic resonances through the bulk of the piezoelectric substrate via actuation across opposing electrodes [109]. The choice of propagation mode directly determines the device’s compatibility with liquid environments. Thickness extensional modes (longitudinal modes) involve particle displacement parallel to wave propagation. While they achieve high Q-factors in air, they suffer from severe acoustic radiation damping and viscous coupling in liquids [110]. Transverse thickness-shear (TS) modes utilize surface-parallel shear displacements to minimize compressional radiation into the fluid. This mechanism maintains high Q-factors in aqueous environments and underpins the operation of QCMs and shear-mode FBARs [45]. Similarly, lateral extensional or contour modes exploit in-plane membrane vibrations to suppress acoustic damping in viscous biological fluids [111].

Among BAW architectures, QCMs (typically utilizing AT-cut quartz at 5–30 MHz) are widely established for low-frequency gravimetric sensing. Practical applications include the rapid detection of *Salmonella typhimurium* in food matrices [112] and gold-nanoparticle-amplified assays for prostate-specific antigen (PSA) [113]. Moving into the 1–10 GHz regime for high mass sensitivity requires ultrathin piezoelectric films (e.g., AlN or ZnO), typically deployed in FBAR and solidly mounted resonator (SMR) configurations [114,115]. Air-gap FBARs offer excellent baseline Q-factors, enabling the trace-level detection of cancer biomarkers such as human PSA and carcinoembryonic antigen (CEA) using aptamer-modified interfaces [116]. SMRs achieve structural robustness for fluidic integration by employing Bragg reflectors. These resonators demonstrate stable in-liquid performance for quantifying human IgE [117] and function effectively as high-frequency (1.3 GHz) gravimetric biosensors [118].

For fluid manipulation, thick PZT-on-silicon or glass resonators generate macroscopic standing waves for high-throughput, continuous-flow acoustophoresis [37]. The boundary between surface and bulk waves can be intentionally bridged. Surface-reflected bulk waves can be generated by patterning traditional IDTs on piezoelectric substrates with an intermediate wavelength-to-thickness ratio (1 < *λ*/*h* < 3), where *h* is the substrate thickness, and *λ* is the acoustic wavelength. This hybrid architecture utilizes a SAW-like planar layout but reflects bulk acoustic energy off the bottom boundary of the thinned substrate, making it highly effective for rapid fluid jetting and micromixing [82,119].

In contrast to these hybrid configurations, pure bulk architectures, such as TS-mode BAWs, strictly utilize thickness-shear vibration modes. This transverse motion allows them to exhibit significantly lower energy dissipation and higher Q-factors under viscous liquid loading compared with traditional unguided Rayleigh SAWs [39]. However, these system-level comparisons require strict qualification. The relative stability and performance of BAWs versus SAWs depend heavily on the specific operational window. Key variables include the excitation frequency (e.g., <500 MHz for typical SAWs versus 1–10 GHz for FBARs), the substrate’s acoustic impedance, mode purity, electrode configuration, and the complex viscoelasticity of the liquid load [120]. Direct comparisons also require clearly defined metrics: performance evaluation based on absolute gravimetric sensitivity (expressed in Hz·cm^2^/ng to normalize the active area), Q-factor retention in liquids (e.g., Q > 500 for TS-mode BAWs versus Q < 100 for traditional R-SAWs), insertion loss (e.g., <−15 dB), or the ultimate limit of detection (spans from nM to fM) [121,122].

### 2.4. Transduction Strategies in Acoustofluidic Platforms

#### 2.4.1. Optical Sensors

**Figure 6:**
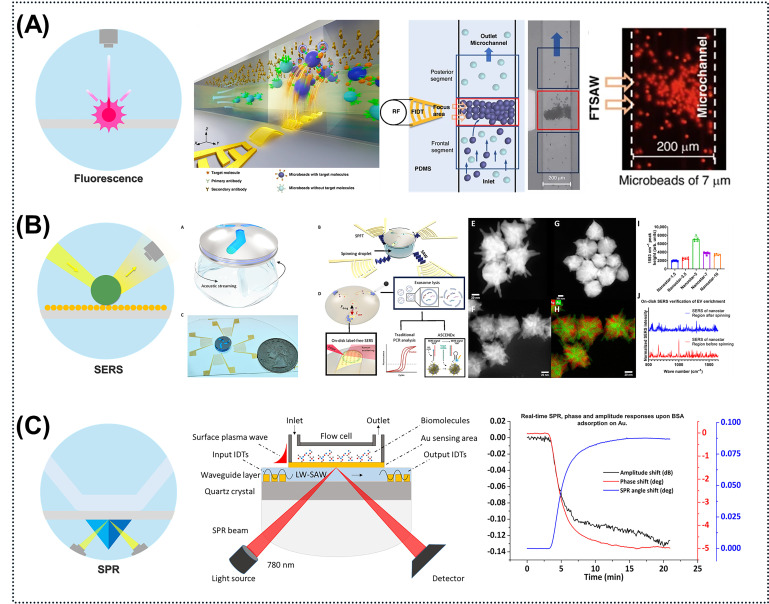
Acoustofluidic-enhanced optical biosensing modalities. (**A**) Integrates focused traveling surface acoustic waves (FTSAW) into a microfluidic chip to rapidly concentrate the microbeads precaptured with human IgG, verifying binding efficiency through fluorescence detection and improving sensing speed to the subsecond level [127]. (**B**) Utilizing an acoustofluidic disc on a spinning droplet to isolate exosomes, coupled with bimetallic plasmonic nanostars for label-free SERS detection [23]. (**C**) SPR: Simultaneous monitoring of SPR reflected light intensity and SAW phase/energy on a LiNbO_3_ substrate for the real-time analysis of biomolecular adsorption [128]. “Figures reprinted with permission (Ref. [127] Copyright 2024 Microsystems & Nanoengineering; Ref. [23] Copyright 2024 Science Advances)”.

Optical sensing modalities—such as fluorescence, surface-enhanced Raman scattering (SERS), and surface plasmon resonance (SPR)—offer high intrinsic analytical sensitivity. However, their practical performance is fundamentally constrained by diffusion-driven mass transport, which dictates long incubation times and yields low local analyte concentrations at the sensor interface. The integration of active acoustofluidic manipulation directly circumvents these kinetic bottlenecks (Figure 6). By employing ARF to spatially concentrate targets into dense clusters or leveraging ASF to rapidly homogenize reagents and disrupt diffusion boundary layers, these hybrid platforms dramatically accelerate detection speeds and enhance the overall SNR [8].

In fluorescence-based assays, acoustic fields physically amplify the optical signal by rapidly sweeping dispersed fluorescently tagged targets into highly localized focal points. For instance, coupling SAW-induced enrichment with metal-enhanced fluorescence effectively bypasses diffusion limits, enabling the rapid and direct quantification of cancer biomarkers from complex matrices [92]. Similarly, standing SAW (SSAW) microchips can actively concentrate functionalized fluorescent capture beads, multiplying the local emission intensity for multiplexed cytokine detection [8]. Beyond static enrichment, dynamic systems such as the “ELISAW” platform utilize SAW-driven microvortices for continuous reagent mixing within microwells, reducing standard fluorescent ELISA incubation times from hours to minutes [123].

For SERS, which provides label-free molecular fingerprinting, the primary physical limitation is the requirement for analytes to physically adsorb onto highly localized plasmonic “hotspots.” Acoustic streaming efficiently resolves this issue by actively and directly pumping biomarkers into these nanostructured regions. Recent advancements include bimodal biosensors that synchronize SAW-driven spatial enrichment with dual fluorescence–SERS readouts for detecting Alzheimer’s disease biomarkers [19,124]. Furthermore, acoustofluidics facilitates the single-step synthesis and assembly of plasmonic silver nanoparticles, producing highly uniform SERS substrates for portable bactericide detection [125]. Naquin et al. [23] recently leveraged SAW-driven helical vortices to rapidly separate and concentrate exosomes directly from clinical plasma, enabling the label-free SERS quantification of circulating microRNA biomarkers for cancer diagnostics.

Finally, SPR biosensing is notoriously bottlenecked by the rapid formation of a target depletion zone immediately adjacent to the functionalized metallic interface. Acoustic microvortices possess the sheer hydrodynamic force to penetrate and disrupt this stagnant boundary layer, continuously replenishing the analyte supply. Modern SAW-enhanced SPR platforms exploit this continuous fluidic recirculation to enable the real-time, high-affinity analysis of biomolecular binding kinetics. For example, integrated SAW-grating SPR biosensors actively refresh the fluidic volume at the sensing interface, preventing target depletion [126]. When combined with localized plasmonic enhancement, this active mass transport rapidly drives nanoparticle-tagged nucleic acids to the sensor surface to achieve the ultrasensitive detection of viral RNA [22]. By substituting passive molecular diffusion with active acoustic mass transport, these hybrid optical platforms realize unprecedented reaction kinetics and analytical sensitivity.

#### 2.4.2. Mass-Based Sensors

Mass-based, or gravimetric, biosensors exploit the mass loading effect. When target analytes—such as proteins, cells, or nucleic acids—bind specifically to a functionalized sensor surface, the accumulated mass induces a measurable shift in the acoustic wave’s resonance frequency or phase [121]. This fundamental mechanism enables the real-time, label-free monitoring of biomolecular binding kinetics.

Transducer selection is dictated by inherent trade-offs among analytical sensitivity, structural robustness, and fabrication complexity. Traditional QCMs operate in the low-megahertz regime (5–30 MHz), providing exceptional baseline stability and low fabrication costs for large targets, though they are inherently constrained by moderate absolute mass sensitivity [129,130]. FBARs push operating frequencies into the gigahertz spectrum, achieving unprecedented mass resolution at the picogram-to-femtogram scale [131]. SMRs are frequently deployed to mitigate the mechanical fragility inherent to free-standing FBAR membranes; these architectures utilize acoustic Bragg reflectors to impart structural rigidity without sacrificing high-frequency resonance. Complementing these bulk-driven architectures, SH-SAW and Love-wave configurations bridge the gap between high gravimetric sensitivity and simplified planar integration. By strictly confining acoustic energy to the sensing interface, they deliver robust responsiveness to surface mass variations within complex biological matrices while remaining highly compatible with planar microfluidic integration [132].

Recent clinical translations of these architectures include the rapid, label-free assessment of cardiovascular disease risk factors in point-of-care (POC) settings [15], ultrasensitive, high-frequency detection of neurological biomarkers such as GFAP [133], and direct biomarker screening within complex physiological fluids (e.g., human saliva) by utilizing Love-wave sensors [78]. Furthermore, SH-SAW platforms have proven highly effective for precisely quantifying the physical properties and size distributions of extracellular vesicles [15,134].

Despite their exceptional intrinsic sensitivities, passive gravimetric sensors are severely bottlenecked by slow diffusion kinetics and the rapid formation of target depletion zones immediately adjacent to the solid–liquid boundary. The active integration of acoustofluidics effectively resolves these mass transport limitations. By generating localized acoustic streaming, these integrated platforms continuously disrupt the stagnant boundary layer and convectively drive suspended analytes directly toward the functionalized resonator surface. This active hydrodynamic transport dramatically accelerates binding kinetics, thus significantly reducing the time-to-result for rapid pathogen and biomarker detection [123,127].

#### 2.4.3. Electrochemical Sensors

Electrochemical biosensors transduce biochemical interactions into measurable electrical signals, utilizing metrics such as amperometric current, potentiometric voltage, or impedance. However, within miniaturized microfluidic architectures, their baseline performance is fundamentally constrained by slow diffusion kinetics. This passive transport mechanism rapidly generates a target-depleted boundary layer, severely impeding the active flux of analytes to the electrode surface [127].

The integration of active acoustofluidics effectively resolves this interfacial barrier. SAW-induced acoustic streaming acts as a powerful localized hydrodynamic actuator. By generating rapid microvortices, this acoustic actuation actively disrupts the stagnant diffusion boundary layer. The convective mass transport significantly accelerates the analyte rate and enhances the electron transfer kinetics at the working electrode. As a consequence, this physical intervention improves analytical sensitivity and drastically reduces sensor response times while effectively preserving the structural integrity and bioactivity of delicate biomolecules.

The clinical utility of this synergy is evident in acoustofluidic-enhanced amperometric immunosensors, which enable the highly sensitive detection of Alzheimer’s disease biomarkers. By actively driving amyloid-beta (Aβ) and tau proteins directly from clinical plasma toward the sensor interface, these platforms achieve an ultralow limit of detection (LOD) of 62.3 fg/mL [124]. Similarly, coupling electrochemical impedance spectroscopy with acoustic streaming enables the rapid, label-free detection of pathogens. This active spatial concentration of analytes at the sensing interface lowers empirical detection limits by two to three orders of magnitude [135].

**Figure 7:**
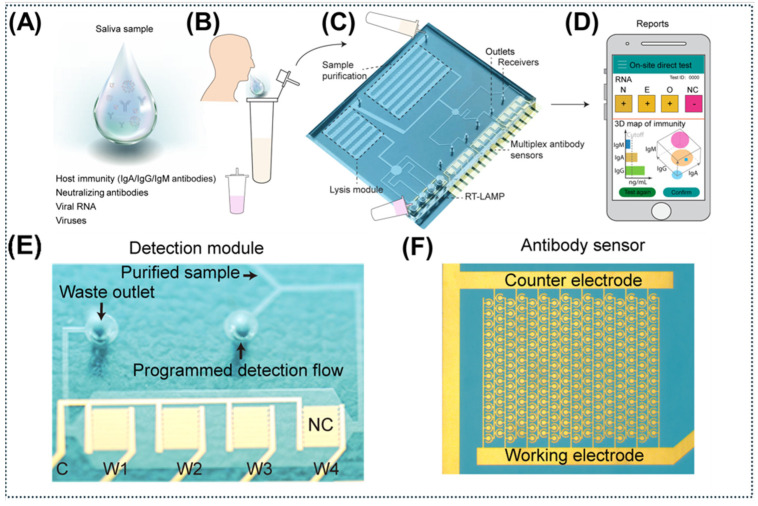
Complete sample-to-answer workflow of the integrated AIMDx acoustofluidic platform. (**A**) Key biomarkers, including host antibodies and viral genetic material, are present in clinical saliva samples. (**B**) Procedure for self-directed saliva sample collection and reagent preparation. (**C**) Photograph of the integrated AIMDx chip, highlighting its modules for sample purification, viral lysis, RNA detection, and antibody detection. (**D**) Comprehensive detection report. (**E**) Schematic of the sensor architecture detailing the working and counter electrodes. (**F**) Comprehensive diagnostic report displaying colorimetric RT-LAMP results for viral RNA and quantitative 3D profiling of host immunoglobulins (IgA, IgG, and IgM) [22]. “Figures reprinted with permission [22]. Copyright 2025 Science Advances”.

The integration of acoustic actuators with screen-printed electrodes yields highly scalable, high-throughput POC platforms. This configuration effectively reduces fluidic mixing times and completed diagnostic assay in 8 min [13]. The recently developed Acoustofluidic Integrated Molecular Diagnostics (AIMDx) chip synchronizes acoustic sample purification with an amperometric sensor array to directly analyze raw saliva. Th device utilizes acoustic streaming shear forces to mechanically dissociate target antibodies (e.g., IgA) from interfering with mucoadhesive proteins (mucins). This reagent-free sample processing enables the simultaneous, highly sensitive multiplexed detection of viral RNA and host immunoglobulins, achieving an LOD as low as 15.6 pg/mL [22]. The comprehensive sample-to-answer workflow and structural design of this AIMDx platform are detailed in Figure 7.

### 2.5. Design Operational Strategies for Acoustofluidic Biosensors

As detailed in the preceding sections, the successful development of an acoustofluidic biosensor relies on the synergistic integration of acoustic manipulation (Section 2.3) and downstream transduction modalities (Section 2.4). However, the optimal device configuration is not one-size-fits-all; it is strictly governed by the target analyte’s size, the desired transport dynamics (e.g., kappa factor κ), and the complexity of the sample matrix.

To bridge the gap between fundamental physics and practical biomedical applications, Table 3. presents a generalized decision-making map. This matrix translates theoretical thresholds—such as the critical particle diameter ($D_{c}$) and the dimensionless size parameter (κ)—into actionable design rules. By mapping specific target classes (from millimeter-scale cells to nanoscale biomarkers) to their dominant physical mechanisms and preferred transducer architectures, this decision map provides a framework for designing application-specific acoustofluidic platforms before entering clinical validation.

**Table 3:** Operational strategies for acoustofluidic biosensors.

Target Scale & Examples	Dimensionless Parameters	Dominant Mechanism	Transport Dynamics	Primary Application
Microscale (1–30 µm, e.g., CTCs, *E. coli*, *Salmonella*, RBCs, WBCs)	κ>1 $d_{P}>D_{c}$	ARF-Dominant	SSAW/TSAW/BAW	Continuous-flow acoustophoresis for label-free size/compressibility sorting; high-shear streaming for rapid on-chip cell lysis (e.g., AIMDx).
Nanoscale (30–200 nm, e.g., Exosomes (sEVs), SARS-CoV-2, HIV, H1N1)	κ≪1 $d_{P}$ *≈* $D_{c}$	Transition Regime	R-SAW/SWANS/SRBW	In-droplet mixing, target enrichment, and continuous concentration. Acoustic micro-vortices for spatial enrichment; carrier-bead trapping to re-establish ARF dominance; layer-parameter for size profiling.
Molecular (2–30 nm, e.g., proteins, DNA/RNA)	κ≪1 $d_{P}<D_{c}$	ASF-Dominant	Love wave/SH-SAW	Dynamic mixing to disrupt depletion boundary layer, accelerating binding kinetics (e.g., ELISAW); label-free gravimetric or bimodal (Optical/SERS) detection.
Ultra-small (<1 nm, e.g., heavy metals (Pb^2+^), dopamine, uric acid, toxins)	κ≪1 $d_{P}\ll \lambda$	ASF-Dominant Acousto-electric effect & Mass Amplification	UHF SAW (>1 GHz)/SMR/FBAR/Love wave	Acoustoelectric coupling (pH/conductivity shifts); mass-amplifying tags (AuNPs, Aptamers) to artificially increase mass load.

Note: $d_{P}$ represents the particle diameter, λ is the acoustic wavelength, κ is the dimensionless Helmholtz number ($\kappa =\pi d_{p}/\lambda$), and $D_{c}$ represents the critical particle diameter.

## 3. Multiscale Acoustofluidic Biosensing: From Molecular Detection to Disease Diagnostic

### 3.1. Target-Oriented Acoustofluidic Biosensors

Table 4 summarizes the performance metrics of acoustofluidic biosensors across a spectrum of multiscale biological and chemical targets, ranging from whole pathogens to molecular biomarkers, for the systematic evaluation of the translational potential of these platforms.

#### 3.1.1. Pathogens: Bacteria & Viruses

The rapid and precise detection of infectious pathogens is paramount for global disease. Traditional diagnostic methodologies—such as cell culture, PCR, and ELISA—are time-consuming, labor-intensive, and require sample preparation and laboratory infrastructure [136]. Acoustofluidic technologies have the potential to address these translational bottlenecks by providing contactless, highly integrated platforms suitable for POC diagnostics [137].

Acoustofluidic biosensors enable detection in complex matrices by integrating target enrichment, nonspecific binding removal, and rapid on-chip lysis, thus improving assay times from days to minutes (15–30 min). To overcome mechanically robust bacterial and viral defense envelopes, high-intensity acoustic actuation generates localized shear stresses sufficient to rapidly rupture cell membranes; for instance, the AIMDx chip achieves complete viral lysis in 2 min [22]. Acoustic purification—utilizing subwavelength Gor’Kov potential wells, standing SAWs, or acoustic nanosieves—selectively traps target pathogens while avoiding contaminants. This achieves purities (e.g., 95.65% for *E. coli*) and significantly amplifies sensor performance [119]. Other integrated platforms have demonstrated robust clinical utility, achieving LODs of 10 CFU/mL for *Salmonella* [138] and 8 CFU/mL for *E. coli* when utilizing a 1 GHz EMPAS aptasensor [139].

The direct acoustic manipulation of nanoscale viral targets (20–200 nm) presents a fundamental physical challenge that illustrates the force balances previously outlined in Section 2.3. At this submicron scale, the ASF induces viscous drag (which scales linearly with particle radius, r) and inherently dominates the F_pARF_ (which scales with particle volume, r^3^) [140]. To circumvent this ARF limitation, viral manipulation frequently employs indirect carrier-bead trapping methodologies—successfully demonstrated for dengue, Ebola, and HIV [141,142]—which artificially increase the effective target volume to reestablish ARF dominance to concentrate.

As alternatives, direct-trapping modalities exploit complex acoustic fields, such as nondiffractive Bessel beams and interparticle SWANS, to overcome ASF [143,144]. By employing acoustic purification strategies with rapid on-chip lysis, platforms such as AIMDx significantly enhance the sensitivity of viral RNA detection. By achieving viral enrichment efficiencies exceeding 95% in 2 min of lysis and ensuring rapid assay completion, AIMDx offers a compelling and robust solution for decentralized POC diagnostics in resource-limited settings [22]. Furthermore, high-frequency acoustic biosensors enable the label-free detection of viral antigens and SARS-CoV-2 antibodies, achieving picogram-to-femtogram LODs [145]. In addition to concentration, the hydrodynamic principles of ASF uniquely enhance assay specificity. Localized R-SAW microstreaming has been exploited to dynamically wash sensor surfaces, rapidly removing up to 94% of interfering, nonspecific proteins without the need for chemical eluents [95]. Recently established standardized protocols for bioparticle isolation, acoustofluidic pathogen platforms have matured into highly potential diagnostic systems [88].

#### 3.1.2. Multi-Scale Cellular and Antigen Biomarkers

Acoustofluidic biosensors exhibit potential versatility across a dimensional spectrum, encompassing small molecules (<1 nm) up to whole cells (10–30 um). Utilizing this multiscale landscape requires optimizing the acoustic mode, operating frequency, and transduction mechanism to the specific physical suitable of the target.

UHF biosensors operating at GHz frequencies are deployed to achieve the exceptional mass sensitivity required for small molecules and ions (<1 kDa). For example, a label-free 1.28 GHz R-SAW resonator can successfully resolve the binding kinetics of small-molecule biotin to immobilized streptavidin receptors at concentrations as low as 104 pM [121]. To compensate for the inherently reduced active sensing area of UHF devices, they must be tightly integrated with microfluidic confinement to ensure efficient analyte delivery.

For large, clinically relevant targets such as proteins and biomarkers (5–150 kDa, 2–10 nm), SAW and Love-wave sensors (50–500 MHz) effectively balance mass sensitivity and biocompatibility. In cardiovascular risk assessment, a ~57 MHz microfluidic SAW device achieved a 4 ng/mL LOD for C-reactive protein (CRP), uniquely measuring mass loading via amplitude variations (insertion loss) rather than traditional frequency shifts [146]. For diabetic retinopathy screening, a 20 MHz SAW chip combined rapid acoustic particle concentration with immunofluorescence to achieve a 40 pg/mL LOD for LCN1 in tear fluid within 3 min. Its detachable design allows IDT reuse while preventing cross-contamination [7,8]. Furthermore, functionalized SH-SAW sensors have achieved sub-ng/mL LODs for epidermal growth factor in 15 min while maintaining high Q-factors in liquid samples [147].

At the nanoscale, acoustofluidics has fundamentally transformed the isolation and characterization of extracellular vesicles (EVs). A novel label-free methodology utilizes the ratio of acoustic attenuation to velocity shift to directly assess EV size. This acoustic “layer parameter” effectively differentiates distinct EV subpopulations (e.g., CD9 and GPC1) independent of particle concentration [134]. For EV manipulation, platforms such as ASCENDx leverage SAW-induced streaming within microdroplets to function as an “acoustofluidic centrifuge”, yielding 95.8% sensitivity and 100% specificity for miRNA-based colorectal cancer diagnostics [23]. Similarly, the rapid acoustic concentration of nanoparticle-bound exosomes within glass capillaries—from volumes as low as 0.5 μL—can amplify localized fluorescent and SERS emission signatures [19]. To achieve high-resolution sorting, the ANSWER platform generates virtual acoustic pillars to continuously fractionate 60–80 nm miRNA-rich exosomes from larger 90–150 nm protein-rich vesicles, achieving over 96% purity in 10 min [148]. These isolation modalities show potential application in clinical settings for highly reproducible small EV separation while preserving delicate vesicular morphology.

For cellular-scale targets (>1 μm), label-free CTC separation using tilted-angle SSAW achieves recovery rates of 83–90% at 7.5 mL/h throughputs [149]. This acoustic manipulation maintains cell viability above 95%, potentially enabling its application in single-cell sequencing and drug susceptibility testing. Another CTC separation platform utilizes an acoustofluidic standing wave field to separate cells according to their inherent biomechanical properties and isolate intact multicellular CTC clusters, resulting in inherently label-free and epitope-independent cell enrichment. By capturing individual CTCs and heterogeneous clusters, this approach can provide a complete assessment of circulating malignant cells and potentially enhance therapy monitoring [87,150].

#### 3.1.3. Chemical & Molecular Markers in Biomedical Diagnostics

The gravimetric detection of small chemical molecules (<1 kDa) and monoatomic ions presents a challenge for acoustic sensors because of their low mass loading on the transducer surface. To overcome this physical limitation, researchers coupled high-frequency architectures (e.g., FBARs and Love-wave sensors) with highly specific recognition elements—such as enzymes, molecularly imprinted polymers (MIPs), or aptamers—and secondary mass-amplifying tags [151]. For instance, neurotoxic heavy metal ions (e.g., Pb^2+^ and Hg^2+^) can be selectively captured by DNAzymes or aptamers conjugated with gold nanoparticles. These heavy metallic tags increase the local mass condition, effectively translating a molecular-scale binding event into a measurable acoustic frequency shift. This frequency shift value and mass loading variation can be described by $\Delta f=k\Delta mf_{0}^{2}/A$. where k represents the sensitivity constant specific to the SAW transducer and experimental setup, and A is the active area of the sensitive region. By utilizing high-density nanoparticles, the Δm is artificially increased, thereby amplifying the Δf signal to detectable levels for molecular-scale targets [152]. Beyond this application, acoustic platforms have the potential to increase analytical accuracy for clinical metabolites and toxins. In the realm of label-free diagnostics, MIPs provide robust synthetic matrices capable of capturing neurological biomarkers such as dopamine with LODs as low as 0.1 pg/mL [153].

The transduction mechanism for small molecules is not strictly confined to pure mass loading. Acoustic devices can also exploit acoustoelectric coupling—the interaction between the piezoelectric potential of the acoustic wave and the charge carriers in the adjacent medium. For example, a 53.7 MHz Love-wave biosensor quantified uric acid (LOD = 5 µM) by monitoring uricase-catalyzed oxidation; the resulting localized pH fluctuation drastically altered the conductivity of the guiding layer, inducing a pronounced acoustoelectric frequency shift independent of the analyte’s absolute mass [154].

**Table 4:** Performance of Acoustofluidic Biosensors Across Multi-Scale Biological and Chemical Targets.

Target	Specific Target	Size	Acoustic Wave/Tech	Transduction	Volume	LOD/	Ref.
Viruses	SARS-CoV-2	20–200 nm	R-SAW (AIMDx)	Mass-Based	100 µL	15.6 pg/mL	[22]
Viruses	SARS-CoV-2	20–200 nm	R-SAW (Bessel Beam)	Electrochemical	30 µL–2 mL	1.96 × 10^6^ bioparticles/µL (1.13 µg/mL)	[143]
Antigens	CRP	2–10 nm	SH-SAW	Mass-Based	50 µL	4 ng/mL	[146]
Antigens	LCN1	2–10 nm	R-SAW	Optical	4 µL	40 pg/mL	[8]
Toxins	Endotoxin	2–10 nm	SH-SAW	Mass-Based	(N/A)	3.53 ng/mL	[102]
Small Molecules	Uric Acid	<1 nm	Love wave	Acousto-electric	10 µL	0.84 µg/mL	[154]

### 3.2. Disease-Oriented Acoustofluidic Biosensors

Table 5 summarizes the applications of acoustofluidic biosensors for detecting a diverse array of disease-specific biomarkers to illustrate the clinical translational potential of these platforms.

In the field of cardiovascular disease, acoustofluidic biosensors provide rapid, POC capabilities for robust clinical risk assessment. For example, SAW biosensors integrated with microfluidic channels can effectively quantify CRP, a key inflammatory marker, within a 10 min assay. This platform achieves an LOD of 4 ng/mL [146]. Furthermore, a portable SH-SAW POC platform was recently deployed to monitor longitudinal cardiovascular risk following dietary (e.g., almond and oat milk) or statin interventions. This reader accurately quantified apolipoprotein B (ApoB)—a key atherogenic biomarker—from 5 μL of finger-prick blood sample in 1 min [15]. This rapid, label-free, and convenient monitoring is suited for home use, rural clinics, and decentralized pharmacy testing.

In addition to cardiovascular monitoring, acoustofluidics has advanced the detection of neurodegenerative disease, moving diagnostic methods from invasive cerebrospinal fluid sampling to direct blood plasma analysis. An integrated acoustofluidic diagnostic system (ADx) effectively differentiates patients with Alzheimer’s disease from healthy controls and efficiently decreases contaminating background bioparticles, enhancing the SNR. The system can isolate and purify Aβ and tau proteins. After isolation, it uses SERS and electrochemical immunosensors for the detection of ZnO–Ag nanorod arrays and achieves an LOD of 62.3 fg/mL for Aβ protein. Validation through multivariate analysis in clinical plasma samples underscores its profound potential for early, minimally invasive diagnostic screening [124].

With the expansion of POC capabilities for ophthalmic conditions, the rapid and noninvasive screening for diabetic retinopathy has been successfully realized with a detachable SAW using human tear fluid. Its reusable IDT architecture, combined with a disposable cover glass, effectively eliminates biological cross-contamination and reduces per-test costs. This only detachable SAW system requires only 4 µL sample volume, uses a 20 MHz SAW to simultaneously mix and concentrate polystyrene microbeads, and achieves a 3.6-fold amplification in the immunofluorescence signal within just 5 s and an LOD of 40 pg/mL in a total assay time of 3 min. A subsequent clinical validation study showed 99% accuracy for the LCN1 biomarker compared with standard ELISA and ophthalmoscopic imaging [7].

In the realm of infectious diseases, acoustofluidics directly addresses the urgent demand for rapid, decentralized screening. For HIV diagnostics, a smartphone-connected, dual-channel SAW biosensor operating at 251.5 MHz has been clinically validated in resource-limited settings. Analyzing only 6 µL of plasma, the device delivered diagnostic results in 60 s, achieving 100% sensitivity for anti-gp41 antibodies and specificity across 133 patient samples [24]. The COVID-19 pandemic further sped up the development of viral detection platforms, yielding SH-SAW biosensors capable of quantifying anti-SARS-CoV-2 nucleocapsid IgG antibodies in under 10 min [145,155].

As researchers progress toward fully autonomous POC systems, they have created a 3D-printed acoustic platform that combines acoustic wave sensors with loop-mediated isothermal amplification (LAMP) and smartphone operation. This portable device successfully detected *Salmonella* directly from 2.5 µL of crude human samples—including whole blood, saliva, and nasal swabs—reaching an LOD of 4 × 10^3^ CFU/mL within 30 min [156]. With its light weight, affordable hardware, and equipment-free interface, this platform represents a significant stride toward realizing decentralized, highly accessible molecular diagnostics in resource-limited environments.

In clinical oncology, a novel dual-mode SAW biosensor has demonstrated enhanced CEA detection directly within viscous human plasma. By applying a 78.5 MHz Rayleigh wave for active acoustic mixing—which overcomes diffusion limits to accelerate antigen–antibody binding kinetics—with a 125 MHz Love-wave mode for highly sensitive gravimetric detection, this platform significantly reduces assay saturation time and improves the LOD to 4.68 ng/mL [157]. Its dual-delay-line configuration effectively mitigates the thermal noise and environmental drift induced by Rayleigh-wave acoustic heating, thus ensuring measurement accuracy.

Looking ahead, the evolution of acoustic biosensors toward clinical-grade oncological platforms will be driven by the multiplexed detection of biomarker panels to bolster diagnostic accuracy and the seamless integration of upstream acoustofluidic sample preparation (e.g., rare cell separation and exosome extraction) for the direct analysis of complex biofluids. Supported by a growing emphasis on rigorous clinical validation, these label-free, real-time platforms are uniquely positioned to revolutionize the liquid biopsy landscape, particularly for the detection of cancer-related biomarkers, EVs, and circulating tumor cells [158].

**Table 5:** Applications of acoustic biosensors for disease biomarker detection.

Disease Category	Target	Mode	Transduction	Volume	LOD	Ref.
Cardiovascular	CRP	SH-SAW	Mass-Based	50 µL	4 ng/mL	[146]
Cardiovascular	ApoB	SH-SAW	Mass-Based	5 µL	80.1 µg/mL	[15]
Neurodegenerative	Amyloid-β/Tau	R-SAW	Electrochemical/Optical	15 µL	62.3 fg/mL (Aβ)	[124]
Ophthalmic	LCN1	R-SAW	Optical	4 µL	40 pg/mL	[6,8]
COVID	Viral RNA & IgA/IgG	R-SAW (AIMDx)	Electrochemical	100 µL	15.6 pg/mL (IgA)	[22]
HIV	anti-gp41 antibodies	SH-SAW	Mass-Based	6 µL	p24:22.2 μg/mL gp 41:25.5 μg/mL	[24]
carcinoembryonic	CEA	Love wave	Mass-Based	20 µL	4.68 ng/mL	[157]

## 4. Challenges and Emerging Trends

Moving acoustofluidic sensors from laboratory curiosities to field-deployable clinical tools requires overcoming a gauntlet of engineering and economic hurdles. To provide a clear roadmap and avoid conflating current limitations with future solutions, this section stratifies the discussion across three distinct levels: device engineering, application processing, and industrialization.

### 4.1. Device-Level: Thermal Management and Precision Packaging

At the fundamental device level, the main physical constraints are acoustothermal heating and rigid packaging. High-frequency actuation inevitably generates acoustic energy at the thermal boundary layer, creating steep temperature gradients that can damage the bio sample and reagent and trigger signal drift [159,160]. Furthermore, the translation of these prototypes to POC settings is hindered by traditional PDMS packaging, which exhibits high acoustic absorption, rendering the devices inefficient.

The shift toward Micro-Electro-Mechanical Systems packaging standards, such as flip-chip bonding on acoustically rigid silicon, is an emerging trend that aims to overcome the above limitations. In addition, advanced digital conditioning shows potential to solve signal phase drift. As a prominent cross-disciplinary case study, the Adaptive Sampling and Sampling Position Optimization method [161], originally developed for high-precision electrohydraulic systems, has proven highly effective at mitigating ripple interference and inductive hysteresis. Adapting such dynamic phase-correction algorithms to acoustic CMOS readouts could enable high phase resolution in multiplexed diagnostics.

### 4.2. Application-Level: Complex Matrices and Fluidic Control

**Figure 8:**
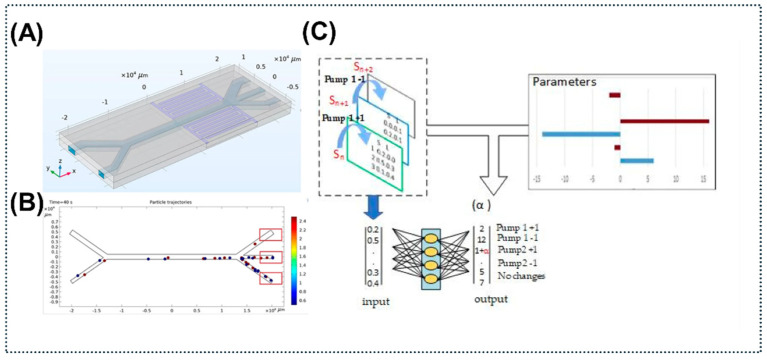
Integration of machine learning in smart microfluidics. The diagram illustrates a reinforcement learning loop where an AI agent continuously adjusts flow rates and acoustic frequencies to enhance microparticle separation efficiency [163]. (**A**) Overview of the microfluidic device, (**B**) distribution of small and large particles in the outlet channels, and (**C**) depiction of AI analyzing the relationship between input and output data.

For application, the primary bottleneck of current platforms is biofouling when handling unprocessed clinical samples. The rapid accumulation of nonspecific proteins from complex matrices, such as whole blood or saliva, poses a risk to blind the acoustic biosensor [162], severely limiting direct clinical usability. The prevailing trend to conquer these matrix effects is the development of a multimodal hybrid biosensor. By coupling acoustic enrichment with SERS or electrochemical detection, these systems physically overcome diffusion limitations and wash out nonspecific binding, thereby cleaning the signal prior to detection [19]. Furthermore, manual fluidic tuning is rapidly being replaced by Artificial Intelligence (AI). A recent study demonstrated the use of machine learning to adjust flow velocity and frequency in real-time [163]. This AI-driven approach creates “Intelligent Microfluidics” capable of automatically adapting to varying sample viscosities (Figure 8).

### 4.3. Industrialization and Clinical Translation

For industrialization, the most daunting barrier is reusability versus disposability. The high cost of high-quality piezoelectric wafers (e.g., LiNbO_3_) prohibits their use as single-use consumables. Meanwhile, the risks of clinical cross-contamination and sterilization requirements make device reusability challenging. The industry’s primary trend to resolve this economic challenge is the adoption of a decoupled modular architecture. Using acoustic superstrates or phononic crystals enables engineers to physically separate an expensive, reusable transducer from a low-cost, disposable plastic microfluidic chip, thereby reducing the cost-per-test.

As an additional case study in future form factors, researchers are increasingly sputtering flexible piezoelectric films (e.g., AlN or ZnO) onto polymer substrates to create conformal, wearable diagnostics for continuous cardiac monitoring [48,164]. The convergence of these modular, flexible platforms with smartphones is poised to fully democratize precision diagnostics [24,165].

## 5. Conclusions and Future Perspectives

Acoustofluidics has advanced biosensing, enabling the field to transition from passive, diffusion-limited assays to active, high-throughput, and label-free diagnostic platforms. Integrating an acoustofluidics platform with optical, electrochemical sensors, and mass transduction strategies allows it to effectively overcome limitations in mass transport and enable sensitive, multiplexed POCT. Furthermore, the incorporation of AI is rapidly transforming these systems from rigid hardware into adaptive, “intelligent” diagnostics. These systems are capable of real-time fluidic optimization and complex data interpretation. Despite these remarkable advancements, the translation of laboratory prototypes into ubiquitous clinical tools requires bridging the gap between engineering innovation. Looking forward, the integration of acoustofluidics with single-cell analysis and acoustofluidics biosensors shows potential for personalized medicine. The advent of flexible piezoelectric materials and miniaturized CMOS readouts is paving the way for wearable acoustic sensors, offering unprecedented capabilities for continuous health monitoring. Owing to their simple, affordable modular designs, acoustofluidic biosensors can make precision medicine accessible in resource-limited areas and strengthen global healthcare.

## Data Availability

No new data were created or analyzed in this study.
